# Seed Pelleting with Calcium Peroxide Improves Crop Establishment of Direct-seeded Rice under Waterlogging Conditions

**DOI:** 10.1038/s41598-017-04966-1

**Published:** 2017-07-07

**Authors:** Junhao Mei, Weiqin Wang, Shaobing Peng, Lixiao Nie

**Affiliations:** 1 National Key Laboratory of Crop Genetic Improvement, MOA Key Laboratory of Crop Ecophysiology and Farming System in the Middle Reaches of the Yangtze River, College of Plant Science and Technology, Huazhong Agricultural University, Wuhan, Hubei 430070 China; 2 Hubei Collaborative Innovation Center for Grain Industry, Yangtze University, Jingzhou, Hubei 434023 China

## Abstract

Poor crop establishment of direct-seeded rice (DSR) is one of the major constraints to wide adoption of DSR, particularly in areas prone to flooding after sowing or where fields are not level. Seed pelleting is an effective, practical and facile technique to enhance crop establishment under unfavorable environmental conditions. To evaluate the effects of seed pelleting on rice germination, seedling growth and associated metabolic events under waterlogging stress, various seed pelleting treatments including formulae, pelleting times (the weight ratio of pelleting agents: rice seeds = 1:1~7:1 (w/w) and CaO_2_ contents were tested in series of experiments. Naked seeds were maintained for comparison as a control. Pelleting treatments with CaO_2_ significantly increased seed germination and seedling growth of DSR under waterlogging conditions compared with pelleted seeds without CaO_2_ and naked seeds. The optimum weight ratios of CaO_2_ to dry seeds were found to be in the range of 0.6:1–1:1 based on seed germination and seedling growth performance under waterlogging conditions. Under waterlogging conditions, high seed germination percentage and vigorous seedling growth of DSR due to seed pelleting with CaO_2_ was associated with an increase in α-amylase activity, but decrease in alcohol dehydrogenase (ADH) and pyruvate decarboxylase (PDC) activities in pelleted seeds.

## Introduction

Rice (*Oryza sativa* L.), a staple food for more than half of the world’s population, is grown on 163.2 million hectares, with an average annual production of 740.9 million tones across the globe^1^. China is the main producer of rice, contributing more than 29% of total global rice production^2^. Therefore, the stability of China’s rice production is an indispensable part of the global food security^3^. In China, traditional transplanted flooded rice is the major planting pattern of rice and nearly 50% of the rice is grown under such conditions^4^. However, in recent years, depleting water resources, labor shortage and increasing labor cost have threatened the sustainability and productivity of transplanted flooded rice systems, suggesting that traditionally transplanted rice is no longer suitable for sustainable development in China^5, 6^.

Taking the advantages of saving water and labor and increasing system productivity, DSR has been believed to be an optimal option for rice production^7^. Tao *et al*. reported that direct-seeded rice was a feasible and sustainable alternative to transplanted rice in Central China based on comparable or even higher yield performances and greater water use efficiency, as well as lower global warming potential and conducive to mechanization^8^.Many countries have shifted from traditional transplanted flooded rice to DSR^9, 10^. At present, 23% of rice is direct-seeded globally^11^. In China, the planting area for direct-seeded rice is increasing rapidly. By 2012, the percentage of direct-seeded rice area to total rice planting area in China increased to 28%^4^.

Nevertheless, poor crop establishment remains a major obstacle for large-scale adoption of DSR, particularly in areas prone to flooding after sowing or where fields are not level^12–14^. Globally, the flood-prone ecosystem accounts for about 9% of total rice lands, but for many countries in South and Southeast Asia, flood-prone rice can represent more than 25% of total rice lands, and the submergence duration varies from 1 to 10 days^15^. In China, 1/10 of the land areas, 33 million ha of farmland, 70% of the agricultural production are threatened by flood stress^16^. The waterlogging after rice seeding would result in seed floating, or the low seed germination rates, as well as uneven and irregular emergence of rice^17^. Furthermore, prolonged duration of flooding would result in wilting and death of the seeds and seedlings, inadequate crop stands, and eventually the reduction of grain yield^18, 19^.

In recent years, various strategies have been employed to improve abiotic stress tolerance during seed germination. Seed pelleting is an effective, practical and facile technique to enhance rapid and uniform emergence, high seedling vigor, and better yields in many field crops particularly under unfavorable environmental conditions^20^. Pelleting is one type of seed coating technology that is defined as the deposition of a layer of inert materials that may obscure the original shape and size of the seed, resulting in the shape and the general size of the raw seed changed, and a substantial weight increase and improved plantability^21, 22^. Ryu *et al*. reported that seed germination and root colonization of sesame were significantly improved when the sesame seeds were pelleted with a combination of clay, vermiculite and talc; consequently the grain yield was increased by 10.2%^23^. Previous research demonstrated that seed pelleting with ammonium molybdate and ferrous sulfate in rhizobium inoculated seeds improved growth parameters of soybean and yield^24^. Asagi *et al*. indicated that pelletized rice seeds with clay or diatomaceous clay increased germination percentage by 43% and 26%, respectively, compared with naked seeds^25^. Guan *et al*. pelleted tobacco seeds using the combined materials with a superabsorbent polymer, poly hydrogel, and salicylic acid enhanced drought-tolerance and significantly improved seed germination, as well as seedling growth^26^. Other studies found that seeds pelleting with calcium peroxide (CaO_2_) promoted germination of rice directly sown into flooded soil, and improved plant growth^27–30^. However, little work has been done regarding the comparative performance of different seed pelleting formulae in enhancing the submergence tolerance of rice and to explore the mechanisms of pelleting-induced stress tolerance. The objectives of the present study were (1) to examine the effects of various seed pelleting formulae with various pelleting times (the weight ratio of pelleting agents: rice seeds = 1:1~7:1 (w/w)) on seed emergence performance under waterlogging conditions during seed germination and (2) to unravel the physiological and biochemical changes in rice seedlings under the influence of seed pelleting and waterlogging stress to obtain a better understanding of pelleting-induced mechanisms.

## Results

### Experiment 1: Rice seed germination and seedling growth between pelleted and naked rice seeds under waterlogging conditions

#### Seed germination

**Figure 1:**
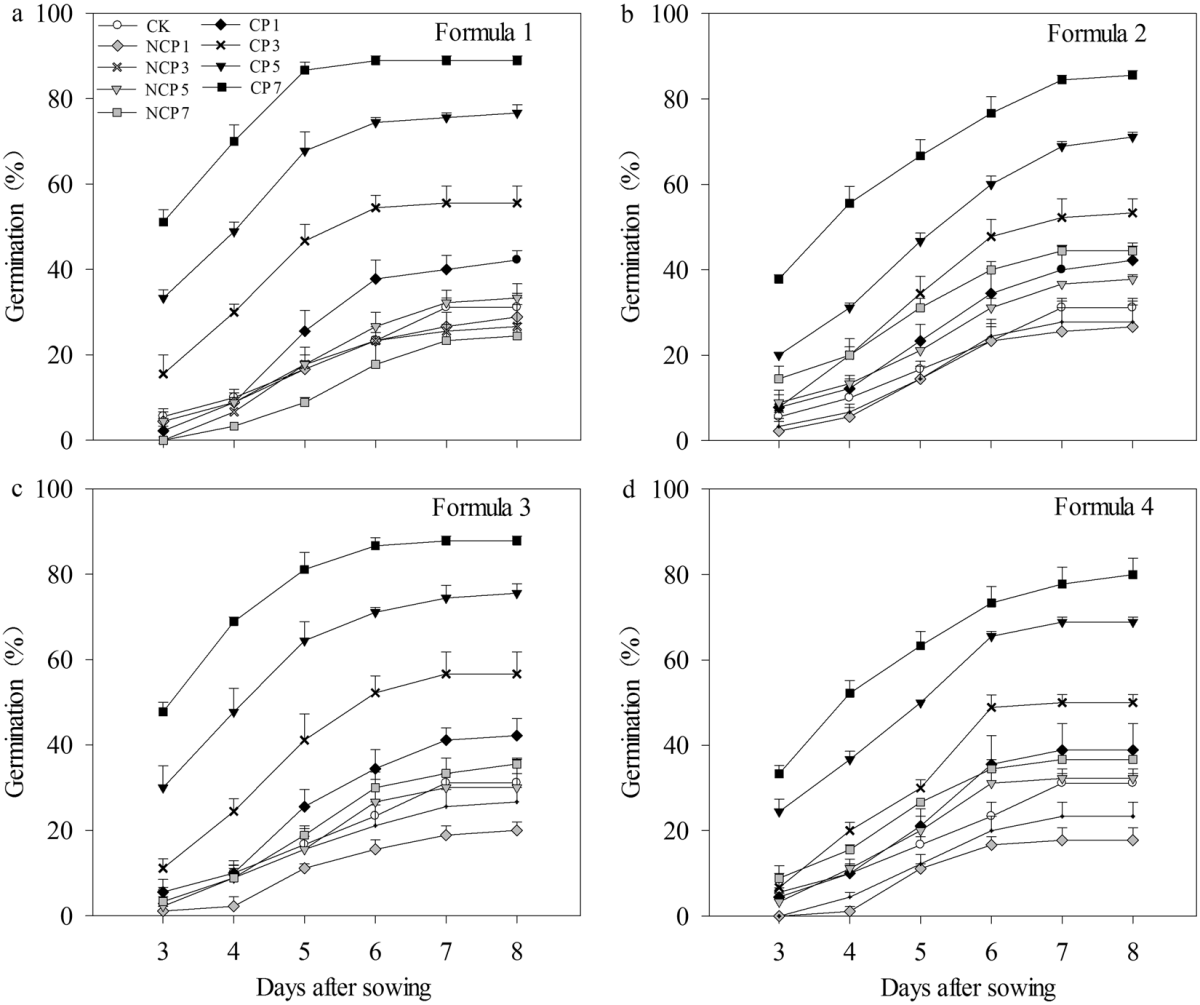
Germination dynamics of pelleted seeds in different formulae with various pelleting times and CaO_2_ contents under waterlogging conditions (1.5 cm water layer) in Experiment 1. (**a**) Formula 1. (**b**) Formula 2. (**c**) Formula 3. (**d**) Formula 4. CK: naked seeds. CP: Calcium peroxide. CP1: Seeds were pelleted by pelleting agents with 10% of CaO_2_ at a pelleting time of 1. CP3: Seeds were pelleted by pelleting agents with 10% of CaO_2_ at a pelleting time of 3. CP5: Seeds were pelleted by pelleting agents with 10% of CaO_2_ at a pelleting time of 5. CP7: Seeds were pelleted by pelleting agents with 10% of CaO_2_ at a pelleting time of 7. NCP1: Seeds were pelleted by pelleting agents without 10% of CaO_2_ at a pelleting time of 1. NCP3: Seeds were pelleted by pelleting agents without 10% of CaO_2_ at a pelleting time of 3. NCP5: Seeds were pelleted by pelleting agents without 10% of CaO_2_ at a pelleting time of 5. NCP7: Seeds were pelleted by pelleting agents without 10% of CaO_2_ at a pelleting time of 7. Seed germination data were recorded from 3 DAS until constant at 8 DAS. Error bars indicate standard error (n = 3).

There were significant (p ≤ 0.05) variations in rice seed germination percentages among different seed pelleting treatments under waterlogging conditions (Fig. 1). The seed germination percentages were significantly higher in the pelleting treatments with 10% of CaO_2_ than those pelleting treatments without CaO_2_ and naked seeds under waterlogging conditions. Furthermore, the seed germination percentage was increased as the pelleting times increased (Fig. 1). Nevertheless, seed pelleting without CaO_2_ was not effective in rice seed emergence under waterlogging conditions. At 8 DAS, the maximum seed germination percentages were above 80% in all formulae, when pelleted with 10% of CaO_2_ at a pelleting time of 7 under waterlogging conditions. While the seed germination percentage of naked seeds was only 31.1%.

#### Seedling growth

Pelleting treatments with CaO_2_ significantly enhanced rice seedling growth under waterlogging conditions compared with pelleted seeds without CaO_2_and naked seeds. Moreover, seedling growth parameters were increased as the pelleting times increased (Table 2). The highest plant growth parameters were observed in pelleting treatments with 10% of CaO_2_ at a pelleting time of 7 (Table 2). On average, seed pelleting with CaO_2_ treatments significantly increased the shoot length, root length, shoot fresh weight, and root fresh weight by 92.99%, 33.49%, 119.39%, and 87.93%, respectively, compared with naked seeds control. While seed pelleting treatments without CaO_2_ did not consistently increase seedling growth parameters compared to the naked seed control. No significant differences in seedling growth parameters were observed among different formulae, as well as pelleting treatments without CaO_2_ and naked seed control.

**Table 1:** Formulae of rice seed-pelleting agents used in the experiments.

Formula	Clay (g)	Talc (g)	Attapulgite (g)	Bentonite (g)	Total weight (g)
1	66.7	33.3	—	—	100
2	—	71.4	28.6	—	100
3	50	50	—	—	100
4	30	60	—	10	100

In experiment 1, Formulae 1–4 were adopted. In experiment 2, Formulae 1 and 2 were adopted. In experiment 3, Formulae 1 and 2 were adopted. In field experiment, Formulae 1 and 2 were adopted.

**Table 2:** The effect of different seed pelleting formulae with various pelleting times on seedling growth parameters of direct-seeded rice under waterlogging conditions (1.5 cm water layer) at 8 DAS in Experiment 1.

Treatment	CaO_2_ content	Pelleting time	Shoot length	Root length	Shoot FW	Root FW
			(cm)	(cm)	(mg seedling^−1^)	(mg seedling^−1^)
Naked seed		0	4.89 ± 0.10 efg	6.50 ± 0.31 bc	14.67 ± 0.59 d	8.08 ± 0.92 e
Pelleting Formula 1	0	1:1	4.28 ± 0.35 g	6.36 ± 0.23 c	13.64 ± 0.97 d	9.68 ± 0.85 de
	0	3:1	4.64 ± 0.30 fg	6.03 ± 0.96 c	14.10 ± 0.89 d	7.72 ± 0.68 e
	0	5:1	5.37 ± 0.16 e	6.43 ± 0.39 c	15.02 ± 0.64 d	8.67 ± 0.79 e
	0	7:1	5.22 ± 0.29 ef	6.40 ± 0.58 c	16.33 ± 1.10 d	8.93 ± 0.62 e
	10%	1:1	8.14 ± 0.19 d	7.16 ± 0.95 bc	29.80 ± 0.68 c	11.64 ± 1.16 cd
	10%	3:1	9.23 ± 0.30 c	8.13 ± 0.49 ab	29.96 ± 1.11 c	13.26 ± 0.30 bc
	10%	5:1	10.42 ± 0.20 b	9.42 ± 0.29 a	35.21 ± 0.94 b	15.12 ± 0.75 b
	10%	7:1	11.69 ± 0.32 a	9.42 ± 0.13 a	41.73 ± 2.15 a	19.07 ± 1.11 a
Naked seed		0	4.89 ± 0.10 d	6.50 ± 0.31 de	14.67 ± 0.59 d	8.08 ± 0.92 c
Pelleting Formula 2	0	1:1	4.53 ± 0.35 d	6.16 ± 0.30 ef	13.60 ± 1.47 d	8.24 ± 1.15 c
	0	3:1	5.12 ± 0.19 d	5.48 ± 0.18 f	13.84 ± 0.75 d	6.88 ± 0.80 c
	0	5:1	5.00 ± 0.12 d	5.90 ± 0.25 ef	14.03 ± 0.44 d	8.30 ± 0.42 c
	0	7:1	6.42 ± 0.25 c	6.55 ± 0.55 de	21.05 ± 0.80 c	12.60 ± 0.80 b
	10%	1:1	7.23 ± 0.42 c	7.15 ± 0.45 d	26.88 ± 0.68 b	13.60 ± 0.61 b
	10%	3:1	8.89 ± 0.42 b	8.13 ± 0.42 c	27.59 ± 2.29 b	13.71 ± 0.84 b
	10%	5:1	10.25 ± 0.20 a	10.10 ± 0.23 a	34.67 ± 1.76 a	17.12 ± 1.29 a
	10%	7:1	11.26 ± 0.67 a	9.17 ± 0.11 b	38.49 ± 3.27 a	18.76 ± 1.30 a
Naked seed		0	4.89 ± 0.10 de	6.50 ± 0.31 b	14.67 ± 0.59 e	8.08 ± 0.92 de
Pelleting Formula 3	0	1:1	4.85 ± 0.21 de	5.56 ± 0.30 b	13.59 ± 1.30 e	8.06 ± 0.23 de
	0	3:1	4.60 ± 0.26 e	5.66 ± 0.50 b	13.49 ± 1.66 e	6.56 ± 0.59 e
	0	5:1	4.92 ± 0.24 de	6.44 ± 0.47 b	15.04 ± 1.54 de	8.63 ± 1.44 cde
	0	7:1	5.76 ± 0.42 d	6.22 ± 0.75 b	19.03 ± 1.35 d	10.87 ± 1.09 cd
	10%	1:1	7.61 ± 0.48 c	6.64 ± 0.41 b	26.18 ± 2.92 c	11.96 ± 2.14 bc
	10%	3:1	8.47 ± 0.34 c	9.22 ± 0.25 a	27.60 ± 1.08 c	15.73 ± 0.54 a
	10%	5:1	9.95 ± 0.56 b	9.72 ± 0.61 a	33.11 ± 0.59 b	15.22 ± 1.29 ab
	10%	7:1	11.52 ± 0.27 a	9.96 ± 0.27 a	40.32 ± 1.71 a	18.44 ± 2.04 a
Naked seed		0	4.89 ± 0.10 e	6.50 ± 0.31 bc	14.67 ± 0.59 c	8.08 ± 0.92 c
Pelleting Formula 4	0	1:1	4.85 ± 0.19 e	5.47 ± 0.27 c	13.91 ± 1.69 c	6.91 ± 0.16 c
	0	3:1	4.66 ± 0.20 e	5.38 ± 0.31 c	13.47 ± 0.70 c	6.95 ± 0.16 c
	0	5:1	5.39 ± 0.36 de	6.59 ± 0.38 bc	15.60 ± 0.92 c	9.82 ± 0.61 bc
	0	7:1	5.87 ± 0.03 d	7.57 ± 0.67 b	17.93 ± 2.67 c	11.79 ± 0.21 b
	10%	1:1	6.98 ± 0.68 c	7.12 ± 0.61 b	23.25 ± 2.18 b	12.37 ± 1.79 b
	10%	3:1	7.75 ± 0.35 c	7.64 ± 0.13 b	26.08 ± 1.87 b	12.77 ± 1.13 b
	10%	5:1	10.42 ± 0.05 b	9.88 ± 0.69 a	36.02 ± 0.88 a	17.40 ± 1.27 a
	10%	7:1	11.31 ± 0.33 a	9.97 ± 0.54 a	37.93 ± 1.72 a	16.89 ± 1.38 a
Formula			ns	ns	ns	ns
CaO_2_ content			**	**	**	**
Pelleting time			**	**	**	**
Formula × CaO_2_ content			*	ns	ns	ns
Formula × Pelleting time			ns	ns	ns	ns
CaO_2_ content × Pelleting time			**	**	**	*
Formula × CaO_2_ content × Pelleting time			ns	ns	ns	ns

Pelleting time is the weight ratio of pelleting agents to rice seeds. Within a column under each pelleting formula, means followed by different letters are significantly different at a 0.05 probability level according to a least significant difference (LSD) test. The naked seeds control were not involved in three-way ANOVA. FW: fresh weight. ** and * denote significance at the 0.01 and 0.05 probability level, respectively. ns: non-significant.

### Experiment 2: Effects of different CaO_2_ contents and pelleting times on seed germination and early seedling growth of pelleted rice under waterlogging conditions

#### Seed germination

**Figure 2:**
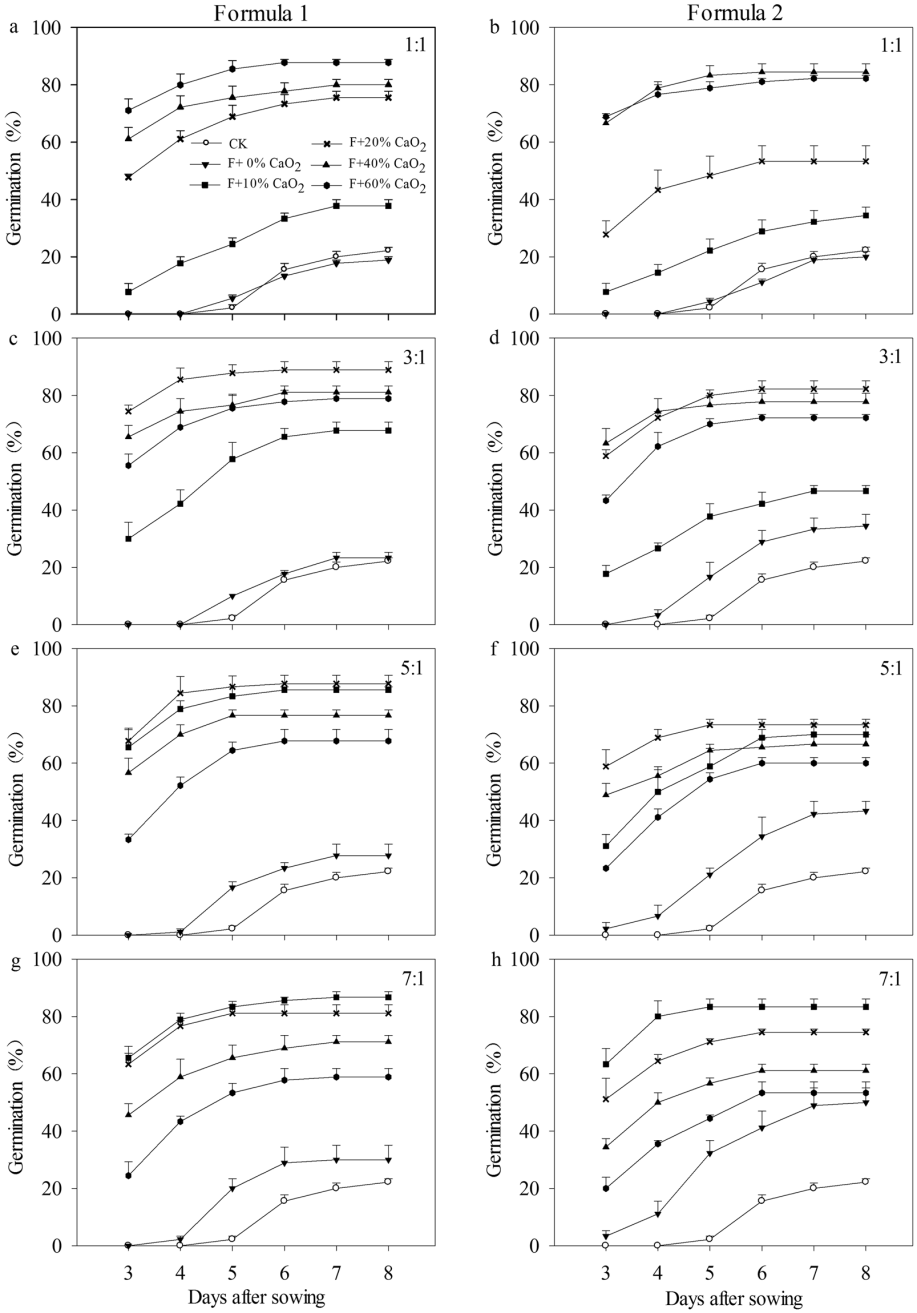
Germination dynamics of pelleted seeds in two formulae with various pelleting times and CaO_2_ contents under waterlogging conditions (1.5 cm water layer) in Experiment 2. Seeds were pelleted in Formula 1 (**a** (pelleting time of 1), **c** (pelleting time of 3), **e** (pelleting time of 5), **g** (pelleting time of 7)) and Formula 2 (**b** (pelleting time of 1), **d** (pelleting time of 3), **f** (pelleting time of 5), **h** (pelleting time of 7)). CK: naked seeds. F + 0% CaO_2_: Seeds were pelleted by pelleting agents without CaO_2_. F + 10% CaO_2_: Seeds were pelleted by pelleting agents with 10% of CaO_2_. F + 20% CaO_2_: Seeds were pelleted by pelleting agents with 20% of CaO_2_. F + 40% CaO_2_: Seeds were pelleted by pelleting agents with 40% of CaO_2_. F + 60% CaO_2_: Seeds were pelleted by pelleting agents with 60% of CaO_2_. Seed germination data were recorded from 3 DAS until constant at 8 DAS. Error bars indicate standard error (n = 3).

All of the pelleting treatments with CaO_2_ significantly enhanced rice seed emergence of rice compared with pelleted without CaO_2_ and naked seed control (Fig. 2). When the weight ratio of pelleting agents to rice seed was 1:1, an increase in CaO_2_ content significantly increased the germination percentage under waterlogging conditions. The maximum germination rates at 8 DAS were 87.8% and 84.4% in formulae 1 and 2, respectively, when the CaO_2_ content in the pelleting agents was increased to 60%. When the pelleting times were 3:1 or 5:1, the highest seed germination percentages were observed at a CaO_2_ content of 20%. When the pelleting time was increased to 7:1, the germination percentages of pelleted seeds decreased with the increase in content of CaO_2_, and the highest germination rates at 8 DAS were 86.7% and 83.3%, respectively, at 10% CaO_2_content in formulae 1 and 2.

#### Seedling growth

All of the pelleting treatments with CaO_2_ significantly enhanced rice seedling growth compared with pelleted without CaO_2_ and naked seed control in both formulae 1 and 2 (Table 3). The highest seedling growth parameters were found at 60% of CaO_2_ content when the pelleting time was 1:1; at 20% of CaO_2_ content when the pelleting times was 3:1 or 5:1; at 10% of CaO_2_ content when the pelleting times was 7:1 in both formulae 1 and 2. The shoot length, root length and their fresh weights increased with the increase in CaO_2_ content, but these parameters were decreased when the weight ratio of CaO_2_ to rice seed was over 1:1. When the pelleting time was increased to 7:1, seedling growth parameters showed continuing downward trends as the content of CaO_2_ increased in formulae 1 and 2 under waterlogging conditions.

**Table 3:** The effect of two seed pelleting formulae with various pelleting times and CaO_2_ contents on seedling growth parameters of direct-seeded rice under waterlogging conditions (1.5 cm water layer) at 8 DAS in Experiment 2.

Treatment	Pelleting time	CaO_2_ content	Shoot length	Root length	Shoot FW	Root FW
			(cm)	(cm)	(mg seedling^−1^)	(mg seedling^−1^)
Naked seed	0		1.92 ± 0.06 j	2.41 ± 0.37 g	6.21 ± 0.27 k	3.61 ± 1.31 gh
Pelleting Formula 1	1:1	0	2.25 ± 0.30 j	2.01 ± 0.91 gh	6.80 ± 0.66 k	3.79 ± 1.32 fgh
	1:1	10%	4.21 ± 0.48 gh	6.17 ± 0.40 cd	14.15 ± 1.80 i	8.63 ± 2.17 de
	1:1	20%	8.00 ± 0.64 c	8.38 ± 1.03 ab	27.22 ± 2.57 cd	17.02 ± 1.42 abc
	1:1	40%	9.22 ± 0.80 b	8.88 ± 0.61 ab	28.39 ± 2.29 cd	18.66 ± 0.47 a
	1:1	60%	10.54 ± 0.63 a	9.58 ± 0.80 a	33.84 ± 2.39 ab	18.36 ± 5.05 ab
	3:1	0	2.47 ± 0.34 ij	3.65 ± 1.14 fg	8.64 ± 1.44 k	4.78 ± 1.40 fg
	3:1	10%	7.83 ± 1.04 c	8.16 ± 1.46 ab	26.67 ± 2.98 cd	14.07 ± 1.68 c
	3:1	20%	9.96 ± 0.71 ab	8.35 ± 0.77 ab	35.18 ± 0.71 a	19.55 ± 0.58 a
	3:1	40%	6.59 ± 0.44 de	7.26 ± 0.97 bcd	25.22 ± 0.85 de	14.57 ± 0.90 c
	3:1	60%	5.05 ± 0.22 fg	5.66 ± 0.80 de	22.29 ± 0.72 ef	10.24 ± 0.79 d
	5:1	0	2.89 ± 0.22 ij	4.25 ± 1.42 ef	10.03 ± 1.24 jk	5.50 ± 2.49 efg
	5:1	10%	7.75 ± 0.43 cd	7.56 ± 0.86 bc	29.95 ± 0.53 bc	15.32 ± 0.85 bc
	5:1	20%	10.40 ± 0.18 a	8.23 ± 0.41 ab	34.77 ± 0.57 a	19.82 ± 2.18 a
	5:1	40%	5.82 ± 0.06 ef	4.21 ± 0.87 ef	20.30 ± 0.89 fg	7.09 ± 0.89 def
	5:1	60%	4.28 ± 0.24 gh	2.39 ± 0.81 g	16.30 ± 0.07 hi	4.81 ± 0.23 fg
	7:1	0	2.59 ± 0.16 Ij	2.43 ± 1.82 g	8.34 ± 0.91 k	3.18 ± 1.74 gh
	7:1	10%	10.25 ± 0.33 ab	9.34 ± 1.10 a	34.60 ± 2.47 a	18.72 ± 3.72 a
	7:1	20%	7.90 ± 0.15 c	7.24 ± 0.69 bcd	26.90 ± 0.58 cd	14.93 ± 1.86 c
	7:1	40%	5.03 ± 0.11 fg	3.19 ± 1.82 fg	18.10 ± 0.90 gh	5.87 ± 2.92 efg
	7:1	60%	3.61 ± 0.19 hi	0.62 ± 0.36 h	13.84 ± 0.73 Ij	1.44 ± 0.75 h
Naked seed	0		1.92 ± 0.06 g	2.41 ± 0.37 gh	6.21 ± 0.27 k	3.61 ± 1.31 jk
Pelleting Formula 2	1:1	0	1.74 ± 0.19 g	1.29 ± 0.90 h	5.04 ± 1.02 k	2.01 ± 0.37 k
	1:1	10%	5.12 ± 0.21 cd	5.19 ± 1.20 de	15.49 ± 1.18 i	9.93 ± 2.52 f
	1:1	20%	7.11 ± 0.23 b	7.48 ± 1.15 bc	21.73 ± 1.02 f	14.62 ± 0.11 de
	1:1	40%	9.43 ± 0.58 a	8.25 ± 1.09 abc	28.26 ± 1.85 cd	16.94 ± 2.44 cd
	1:1	60%	10.02 ± 0.17 a	8.62 ± 0.60 ab	31.46 ± 0.79 ab	20.96 ± 2.30 ab
	3:1	0	3.64 ± 0.78 ef	4.11 ± 1.54 ef	11.74 ± 1.56 j	5.56 ± 2.50 ijk
	3:1	10%	5.69 ± 0.66 c	6.77 ± 1.94 cd	19.75 ± 1.31 fg	11.56 ± 3.96 ef
	3:1	20%	9.60 ± 0.18 a	8.92 ± 0.43 ab	31.08 ± 0.49 bc	19.62 ± 1.31 abc
	3:1	40%	7.12 ± 0.33 b	7.90 ± 0.09 abc	25.03 ± 0.92 d	15.22 ± 0.42 d
	3:1	60%	5.31 ± 0.14 cd	4.93 ± 0.89 e	18.92 ± 0.84 fgh	9.59 ± 1.01 fg
	5:1	0	3.03 ± 0.21 f	4.13 ± 0.82 ef	9.63 ± 0.62 j	5.89 ± 0.68 hij
	5:1	10%	7.43 ± 0.51 b	8.42 ± 0.71 abc	26.09 ± 2.30 de	16.24 ± 4.63 cd
	5:1	20%	10.28 ± 0.32 a	9.43 ± 0.44 a	33.13 ± 0.53 ab	22.91 ± 0.86 a
	5:1	40%	5.32 ± 0.53 cd	4.63 ± 2.08 e	19.37 ± 0.87 fg	9.18 ± 3.22 fgh
	5:1	60%	4.61 ± 0.26 de	2.63 ± 1.07 fg	17.01 ± 0.99 ghi	4.61 ± 2.14 jk
	7:1	0	3.22 ± 0.21 f	4.34 ± 1.22 ef	11.46 ± 0.84 j	6.10 ± 0.81 g hij
	7:1	10%	10.43 ± 0.15 a	9.12 ± 0.20 ab	34.33 ± 0.77 a	18.19 ± 1.91 bcd
	7:1	20%	8.11 ± 0.45 b	8.22 ± 0.72 bc	27.82 ± 1.64 de	16.70 ± 1.32 cd
	7:1	40%	5.66 ± 0.40 cd	4.05 ± 1.83 ef	21.75 ± 1.13 f	8.25 ± 1.52 fgIj
	7:1	60%	3.76 ± 0.22 ef	1.10 ± 0.54 h	15.98 ± 0.36 hi	2.81 ± 1.13 jk
Formula			ns	ns	ns	ns
Pelleting time			*	**	ns	**
CaO_2_ content			**	**	**	**
Formula × Pelleting time			ns	**	**	ns
Formula × CaO_2_ content			ns	ns	*	ns
CaO_2_ content × Pelleting time			**	**	**	**
Formula × CaO_2_ content × Pelleting time			ns	ns	ns	ns

Pelleting time is the weight ratio of pelleting agent to rice seeds. Within a column under each pelleting formula, means followed by different letters are significantly different at a 0.05 probability level according to a least significant difference (LSD) test. The naked seeds were not involved in three-way ANOVA. FW: fresh weight. ** and * denote significance at the 0.01 and 0.05 probability level, respectively. ns: non-significant.

The optimum weight ratio of CaO_2_ to dry seeds was determined based on the highest germination percentage and the best seedling growth, which was achieved under a certain CaO_2_ content at a certain pelleting time. In this study, the optimum weight ratio of CaO_2_ to dry seeds was at 0.6:1–1:1 (a CaO_2_ content × a pelleting time). Adverse effects on seed germination and seedling growth was observed when the ratio was over 1:1.

### Experiment 3: The mechanisms underlying the waterlogging tolerance during seed germination and seedling growth induced by rice seed pelleting with CaO_2_

#### Seed germination and Seedling growth

**Figure 3:**
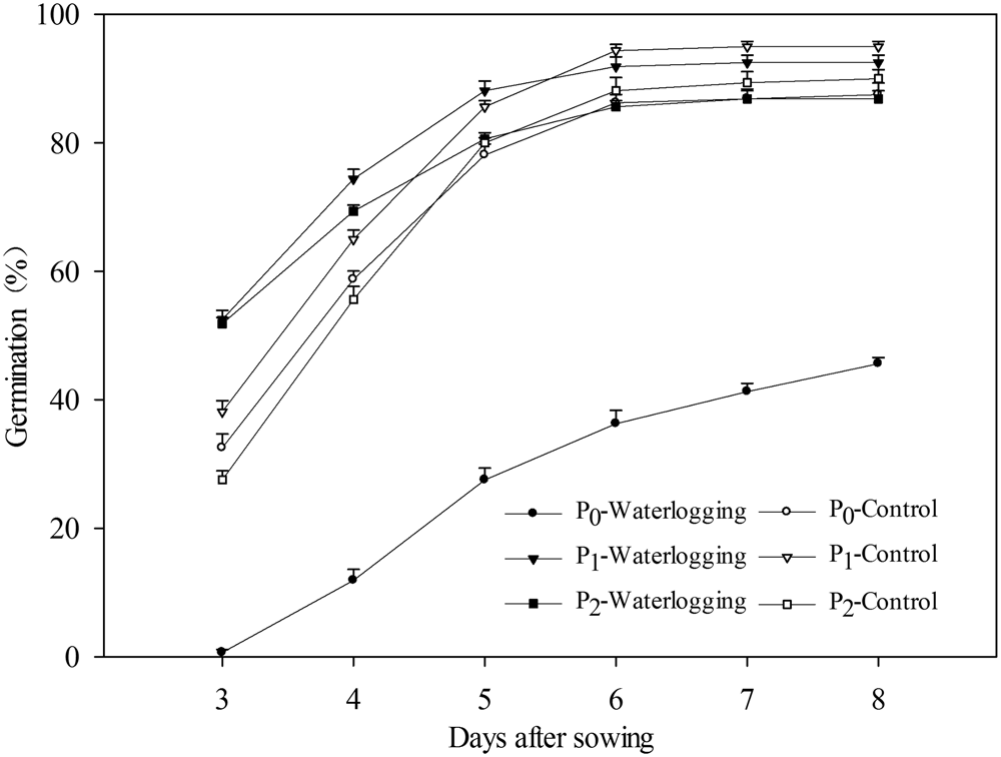
Germination dynamics of pelleted rice seeds in two pelleting treatments under waterlogging (1.5 cm water layer) and normal conditions (without water layer) in Experiment 3. P_0_: non-pelleted naked seeds; P_1_: Seed pelleting time of three with CaO_2_ content of 20% in formula 1; P_2_: Seed pelleting time of three with CaO_2_ content of 20% in formula 2. The seed germination data were recorded from 3 DAS until constant at 8 DAS. Error bars indicate standard error (n = 4).

Results revealed that seed pelleting treatments at a pelleting time of 3:1 with 20% of CaO_2_ significantly (p ≤ 0.05) enhanced seed germination and seedling growth compared with naked seeds under waterlogging conditions, while there were no significant differences in seed germination and seedling growth between pelleted seed treatments and the naked seed control under normal condition (Fig. 3 and Table 4). The germination percentages in the two pelleted treatments under waterlogging conditions and three treatments under normal conditions were above 86.9%, which was significantly higher than naked seeds (45.6%) under waterlogging conditions (Fig. 3).

**Table 4:** The effect of two seed pelleting treatments on seedling growth parameters of direct-seeded rice under waterlogging (1.5 cm water layer) and normal (without standing water) conditions at 8 DAS in Experiment 3.

Water condition	Pelleted Treatment	Shoot length	Root length	Shoot FW	Root FW
		(cm)	(cm)	(mg seedling^−1^)	(mg seedling^−1^)
Waterlogging (Water depth1.5 cm)	P_0_	1.99 ± 0.07 c	1.86 ± 0.14 d	4.79 ± 0.30 e	1.70 ± 0.08 c
	P_1_	7.24 ± 0.13 b	9.73 ± 0.24 c	22.09 ± 0.78 d	18.92 ± 1.11 b
	P_2_	7.09 ± 0.30 b	9.84 ± 0.36 c	21.04 ± 0.47 d	19.00 ± 1.67 b
Control (Without standing water)	P_0_	7.99 ± 0.11 a	12.29 ± 0.04 b	24.35 ± 0.49 c	24.11 ± 0.65 a
	P_1_	8.31 ± 0.09 a	13.11 ± 0.57 a	28.71 ± 0.98 a	25.72 ± 1.01 a
	P_2_	8.13 ± 0.14 a	12.80 ± 0.27 ab	26.13 ± 0.49 b	23.76 ± 0.81 a

Within a column under each water condition, means followed by different letters are significantly different at a 0.05 probability level according to a least significant difference (LSD) test. P_0_: non-pelleted naked seeds; P_1_: Seed pelleting time of three with CaO_2_ content of 20% in formula 1; P_2_: Seed pelleting time of three with CaO_2_ content of 20% in formula 2. FW: fresh weight.

#### Amylase activity

**Figure 4:**
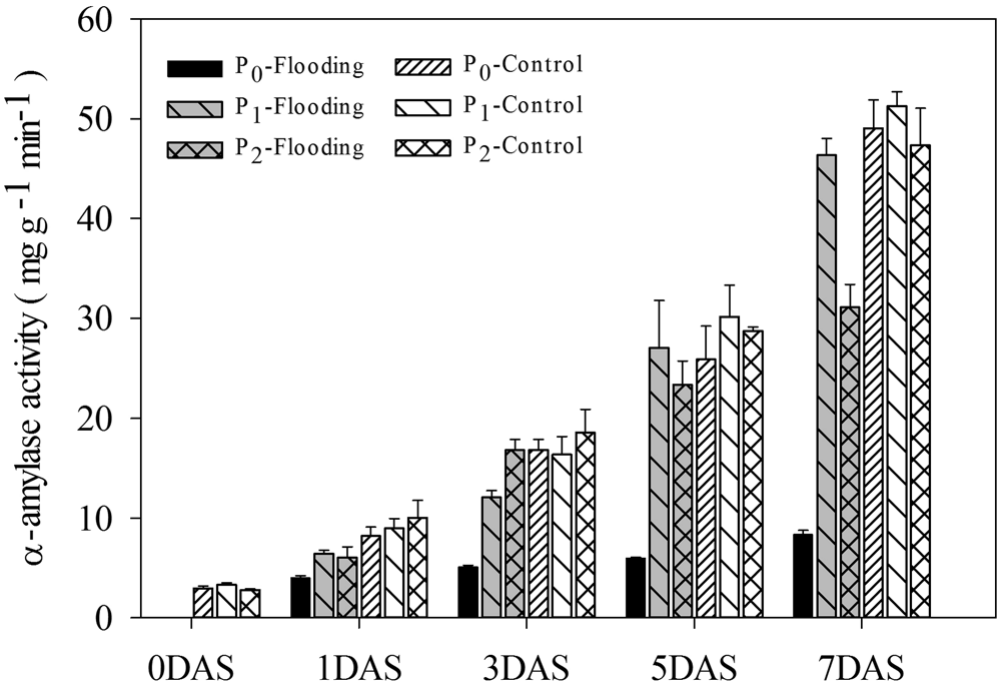
Variations in α-amylase activity of pelleted rice seeds and seedlings in two pelleting treatments under waterlogging (1.5 cm water layer) and normal (without water layer) conditions at 0, 1, 3, 5, and 7 DAS in Experiment 3. P_0_: non-pelleted naked seeds; P1: Seed pelleting time of three with a CaO_2_ content of 20% in formula 1; P_2_: Seed pelleting time of three with a CaO_2_ content of 20% in formula 2. Seeds at 0 DAS were tested under a dry weight basis, while seedlings at 1, 3, 5 and 7 DAS were tested under a fresh weight basis. Error bars above the mean indicate standard error (n = 4).

Waterlogging reduced the α-amylase activity of rice seeds or seedlings compared with normal conditions (Fig. 4). Even under normal conditions, the two pelleting treatments slightly increased the α-amylase activity compared with the naked seed control. Under waterlogging conditions, both of the seed pelleting treatments at the pelleting time of 3:1 with 20% of CaO_2_ content significantly improved the starch metabolism of rice seeds or seedlings. In all treatments, α-amylase activities were progressively increased and achieved the highest values at 7 DAS.

#### ADH and PDC activities

**Figure 5:**
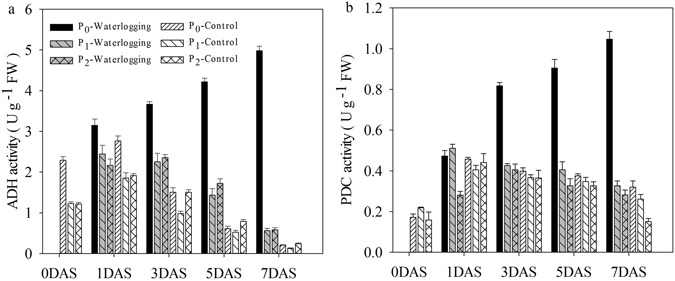
Variations in alcohol dehydrogenase (ADH) (**a**) and pyruvate decarboxylase (PDC) (**b**) activities of pelleted rice seeds and seedlings in two pelleting treatments under waterlogging (1.5 cm water layer) and normal (without water layer) conditions at 0, 1, 3, 5, and 7 DAS in Experiment 3. P_0_: non-pelleted naked seeds; P_1_: Seed pelleting time of three with a CaO_2_ content of 20% in formula 1; P_2_: Seed pelleting time of three with a CaO_2_ content of 20% in formula 2. Seeds at 0 DAS were tested under a dry weight basis, while seedlings at 1, 3, 5 and 7 DAS were tested under a fresh weight basis. Error bars above the mean indicate standard error (n = 4).

ADH activities in rice seeds or seedlings were significantly higher under waterlogging conditions than under normal conditions (Fig. 5a). Under waterlogging conditions, both seed pelleting treatments significantly decreased the ADH activities in rice seeds or seedlings compared with the naked seeds. There were no variations in ADH activities between two seed pelleting treatments under waterlogging or normal conditions. However, seed pelleting treatments didn’t affect the ADH activities in rice seeds or seedlings consistently. The ADH activities in naked seeds under waterlogging conditions continuously increased as seed germination progressed. While in the other five treatments, the ADH activities were progressively decreased as seed germination progressed. Under waterlogging conditions, both seed pelleting treatments significantly decreased the PDC activities in rice seeds or seedlings compared with the naked seeds (Fig. 5b). There were no variations in PDC activities between two seed pelleting treatments under waterlogging or normal conditions.

### Field experiment: Performance of pelleting treatments on seed germination and early seedling growth of pelleted rice under waterlogging conditions in field

#### Seed germination and Seedling growth

**Figure 6:**
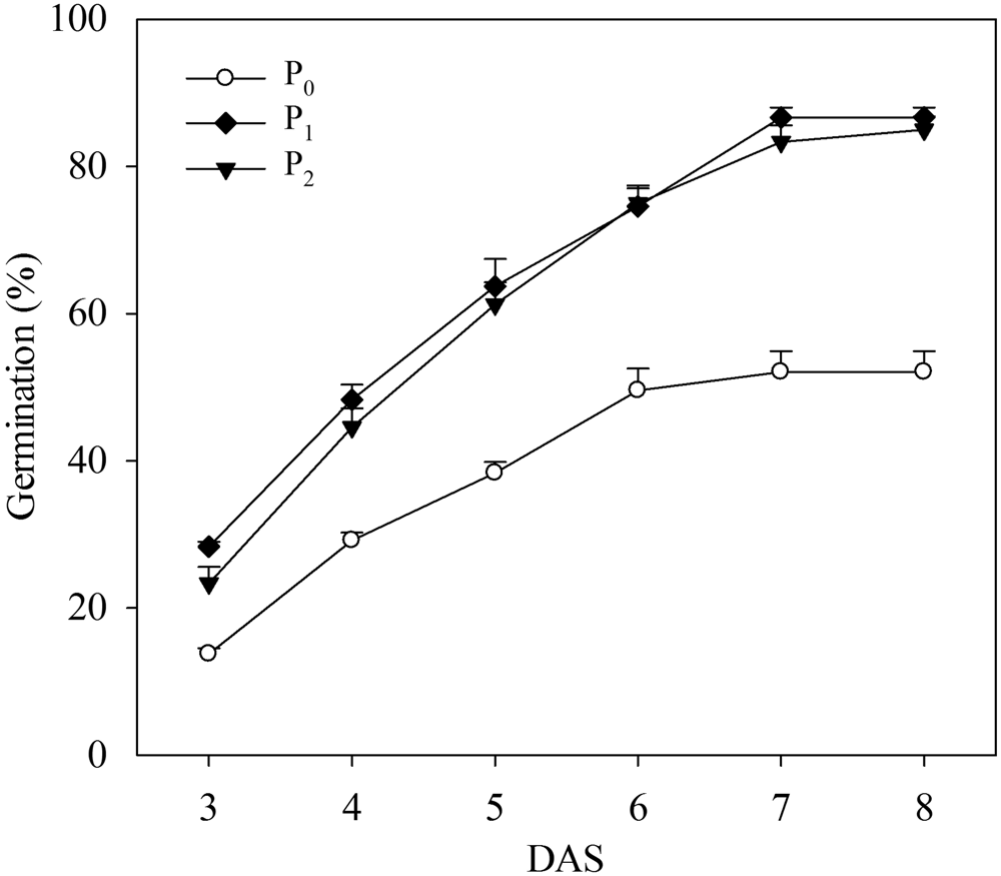
Germination dynamics of pelleted rice seeds in two pelleting treatments under waterlogging conditions in Field. P_0_: non-pelleted naked seeds; P_1_: Seed pelleting time of three with CaO_2_ content of 20% in formula 1; P_2_: Seed pelleting time of three with CaO_2_ content of 20% in formula 2. The seed germination data were recorded from 3 DAS until constant at 8 DAS. Error bars indicate standard error (n = 4).

Seed pelleting treatments at a pelleting time of 3:1 with 20% of CaO_2_ significantly (p ≤ 0.05) enhanced seed germination and early seedling growth compared with naked seeds under waterlogged field conditions, while there were no significant differences between the two seed pelleting treatments (Fig. 6 and Table 5). These were consistent with the results from the growth chamber experiments. On average, the germination percentages of the two pelleting treatments were 33.75% higher than the naked seeds. The shoot length, root length, shoot fresh weight, and root fresh weight in the pelleting treatments were increased by 65.06%, 33.86%, 172.61%, and 182.53%, respectively, compared with naked seeds control.

**Table 5:** The effect of two seed pelleting treatments on seedling growth parameters of direct-seeded rice under waterlogging conditions at 9 DAS in Field Experiment.

Treatment	Shoot length	Root length	Shoot FW	Root FW
	(cm)	(cm)	(mg seedling^−1^)	(mg seedling^−1^)
P_0_	7.37 ± 0.10 b	5.70 ± 0.27 b	7.76 ± 0.29 b	3.55 ± 0.19 b
P_1_	12.18 ± 0.37 a	7.49 ± 0.19 a	21.16 ± 0.66 a	10.56 ± 0.69 a
P_2_	12.15 ± 0.19 a	7.77 ± 0.26 a	21.15 ± 1.29 a	9.50 ± 0.37 a

Within a column under each water condition, means followed by different letters are significantly different at a 0.05 probability level according to a least significant difference (LSD) test. P_0_: non-pelleted naked seeds; P_1_: Seed pelleting time of three with CaO_2_ content of 20% in formula 1; P_2_: Seed pelleting time of three with CaO_2_ content of 20% in formula 2. FW: fresh weight.

## Discussion

Waterlogging seriously impeded seed germination and early seedling growth of DSR, pelleting treatments with calcium peroxide significantly increased seed germination and improved seedling growth. Ellis and Setter reported that the presence of O_2_ in the floodwater was critical for the survival of seedlings under waterlogging conditions^31^. Miro and Ismail reported that rice seeds failed to develop roots and leaves even though they can germinate in submerged conditions^32^. Under waterlogging conditions, the elongations of the coleoptile and mesocotyl were stimulated, which facilitated the exposure to air^33, 34^. However, the fast elongation of the coleoptile and mesocotyl consumes much energy. Sometimes, waterlogging results in plant death if energy reserves are depleted before emergence^35^. In this study, coleoptiles of the pelleted rice seeds with optimal CaO_2_ content first expanded in a thin layer, and later, the coleoptile formed a sturdy morphology and rapid expansion of leaf after contact with air, while the naked seeds seldom germinated.

Baker and Hatton reported that the available oxygen increased as the content of CaO_2_ increased^36^. In this study, increased content of CaO_2_ in the pelleting agents significantly increased the oxygen release to flooding water. In Experiment 2, an increase in the content of CaO_2_ from 10% to 60% at a pelleting time of 3:1, resulted in an increase in the amount of oxygen released from CaO_2_ by 106% (data not shown). Previous reports suggested that elevated oxygen tension may be highly toxic to embryos, root development and root elongation^37^. The chemical reactions of CaO_2_ and water, which usually occur as 2 CaO_2_ + 2 H_2_O → 2 Ca(OH)_2_ + O_2_, produce large amounts of calcium hydroxide and could form an alkaline environment for seeds when pelleted with excessive CaO_2_. In this study, CaO_2_ was added in the pelleting agents increased available oxygen for seed respiration during germination, relieved the anoxia or hypoxia caused by waterlogging conditions. In addition, CaO_2_ may inhibit the proliferation of anaerobic microorganisms, which negatively affect the seed germination. A similar research reported that the germination of seeds coated with streptomycin or a mixture of streptomycin and CaO_2_ was significantly reduced as compared with that of seeds coated with only CaO_2_, which implied that limitation of bacterial pathogens with CaO_2_ might not be as important as O_2_ supply in crop establishment under waterlogging conditions^38^. However, we could not exclude the sterilization effect from CaO_2_ during seed germination in this study. The importance of the antimicrobial effect from CaO_2_ will be evaluated in the future research.

During rice seed germination, oxygen plays an important role on decomposition of reserves in endosperm. While oxygen is limited under waterlogging conditions. CaO_2_ produce large amounts of oxygen in water. Under aerobic conditions, α-amylase activity in rice seedlings was maintained at higher level because of higher expression of *Amy1A* gene compared with that in flooded soils^39^. The α-amylase in rice is likely playing a crucial role for the successful degradation of starch into carbohydrates in the anoxic rice endosperm. Perata *et al*. and Guglielminetti *et al*. indicated that α-amylase activity of the anaerobically germinated rice seeds was markedly lower than that presented in aerobic conditions^40, 41^. Moreover, Saddam *et al*. proclaimed that submergence stress severely reduced α-amylase activity by limiting starch degradation, therefore, rice seed reserves were not well metabolized under submerged conditions^42^. The above findings were consistent with our results that extremely low α-amylase activities were observed for naked rice seeds or seedlings under waterlogging conditions. Pronounced increases in α-amylase activities were recorded in seed pelleting treatments with CaO_2_ under waterlogging conditions.

One of the best characterised plant responses to waterlogging is the metabolic switch from aerobic respiration to anaerobic fermentation. Under submerged conditions, glycolysis and alcoholic fermentation are important processes for the energy production of seeds or seedlings^31, 42^. The two key enzymes involved in glycolysis and alcoholic fermentation are ADH and PDC^43–45^. Magneschi and Perata noted that the inhibited aerobic respiration under submergence conditions resulted in reduced energy production and supply of intermediates needed for seed germination and seedling growth^46^. Several reports had revealed the importance of increases in ADH and PDC activities in acceleration of plant growth and survival under anoxia^47–50^. Tolerance to waterlogging stress during seed germination was expressed as rapid germination, coleoptile elongation, anaerobic respiration to sustain energy supply, and maintenance of the cellular extensibility of the growing embryo^14, 34^. Energy supply is indeed a key factor for rice tolerance to oxygen deficiency. Plants tolerant of anaerobiosis maintain an active fermentative metabolism for a relatively long time^46^, that accelerates carbohydrate consumption that can be expected to increase the energy production^51^, with a 2-3-fold increase in glycolytic flux under anoxia with respect to the aerobic control^50^. Under aerobic conditions, activities of fermentative enzymes, ADH and PDC were decreased to 1/10-1/5 compared to that under anaerobic conditions^52^. In this study, seed pelleting treatments with CaO_2_ decreased the ADH and PDC activities in rice seeds or seedlings compared with the naked seeds under waterlogging conditions. This was because CaO_2_ increased the solubility of O_2_ in water under waterlogging conditions, which may transfer to aerobic respiration in rice seeds or seedlings, thus providing enough energy for the survival or growth of rice seeds or seedlings under submergence conditions.

Conclusively, seed pelleting with CaO_2_ can alleviate the negative effects of flooding stress on rice seed emergence and seedling establishment. High seed germination percentage and vigorous seedling growth due to seed pelleting was associated with an increase in α-amylase activity, but decreases in ADH and PDC activities in pelleted seeds. Seed pelleting with CaO_2_ could be used as a strategy to improve the crop establishment of direct-seeded rice, when seeds suffered from waterlogging soon after seed sowing. However, more exact mechanisms underlying the waterlogging tolerance induced by seed pelleting with CaO_2_ will be addressed in the future. And the effects of seed pelleting treatments need to be wide demonstrated and popularized in practical production.

## Materials and Methods

### Seed source

Seed of a widely grown Indica rice cultivar viz., Huanghuazhan (HHZ, inbred) were obtained from the Crop Physiology and Production Center, Huazhong Agricultural University, Wuhan, China. The initial germination rate of the seeds was >93%. Healthy and filled seeds selected from the same seed lot were used for all of the experiments. Prior to experimentation, seeds were stored in a refrigerator (−4 °C).

### Seed pelleting treatments

Based on previous research, four seed pelleting formulae were adopted for present studies (Table 1). In general, the pelleting formula consists of pelleting agents, including clay, talc, attapulgite and bentonite (all were produced by Shijiazhuang Keying Refractory Material Co., LTD, China) and the adhesive (solution of 1.5% (w/w) polyvinyl alcohol, produced by Sinopharm Chemical Reagent Co., LTD, China). The rice seeds were pelleted with a minitype seed coater (produced by Shanghai Huanghai Drug Testing Instrument Distribution CO., LTD, China) using the following procedures: Firstly, the seeds were quickly soaked in the tap water then air-dried without visible moisture on the surface of seeds. Secondly, 1/4 of the total pelleting agents were slowly placed into the rotate pan of the seed coater (200 mm diameter) at the rotating speed of 45 rpm. Then, the pre-treated seeds were placed into the pan, after then, 1/4 of the total adhesive was sprayed onto the seed surface for 2 min while rolling. Thirdly, another 1/4 of the total pelleting agents were added into the rotate pan with 1/4 of the total adhesive sprayed onto the seed surface, this step was repeated twice. Finally, after all of the pelleting agents and adhesive were added, the pan was continuously rotated for 5 min. The pelleted seeds were then air-dried for 2 days at room temperature. Naked seeds were used as a control.

In Experiment 1, four pelleting formulae were used (Table 1). To evaluate the effectiveness of CaO_2_ on seed germination and seedling growth under waterlogging conditions, two treatments were set in each formula, with 10% (w/w) CaO_2_ (with a purity of 70%) and without CaO_2_, and each treatment included various pelleting times (1, 3, 5 or 7). Because the germination and seedling growth performance of pelleted seeds with CaO_2_ was better compared to naked seeds as well as pelleted seeds without CaO_2_, in Experiment 2, formula 1 (best performance) and 2 (previously reported) were selected. In order to examine the effectiveness of different contents of CaO_2_ on seed germination and seedling growth, based on the proportions of ingredients unvaried, the CaO_2_ content were set as 0%, 10%, 20%, 40% and 60% (w/w) in each pelleting formula with various pelleting times (1, 3, 5 and 7). In Experiment 3, the pelleting formulae were the same as that in Experiment 2, however, only 20% (w/w) of CaO_2_ content at a pelleting time of three was adopted in Experiment 3.

### Experimentation

Growth chamber experiments were carried out in the Crop Physiology and Production Center, Huazhong Agricultural University, Wuhan, China during October 2015 to July 2016. Plastic trays 31.0 cm × 20.0 cm × 12.0 m in size were filled with 7.50 kg of puddled soil which was collected from the field and then soil surface was leveled in trays. In each tray, 30 rice seeds for each treatment were equally sown on the soil surface in experiment 1 and 2 with three replications. In experiment 3, four replications with four trays with 1.5 cm water depth and the other four replications without standing water were used to sample for determinations of α–amylase, alcohol dehydrogenase and pyruvate decarboxylase activity. The experiments were laid out in a completely randomized design. All of the trays were placed in a growth chamber with a 12 h light period and a constant temperature of 25 °C. The humidity during the course of the study was maintained at 60% in the growth chamber. Water was applied to all trays when their moisture content declined. Germination of seeds was recorded on a daily basis according to AOSA until a constant count was achieved^53^. Seeds were considered to be germinated when the hypocotyl length exceeded 2 mm. Seed germination percentage was taken as the ratio of the number of seeds germinated to the total number of seeds sown and was expressed as a percentage. At 8 DAS, 6 seedlings were randomly sampled from each treatment to record their shoot and root lengths. The seedlings were dissected into roots and shoots and their fresh weights were recorded immediately. For determination of α-amylase activity, 1.0 g of dry seeds (0 DAS) and seedlings sampled at 1, 3, 5 and 7 DAS including shoot and root were ground and mixed with 100 ml distilled water, and left for 24 h at 4 °C and then the mixture was filtered with filter paper (Whatman No. 42). The enzyme activity was determined by the dinitro-salicylic acid (DNS) method^54^. To determine alcohol dehydrogenase activity and pyruvate decarboxylase activity, 0.1 g dry seeds (0 DAS) and seedlings sampled at1, 3, 5 and 7 DAS were ground and detected by an alcohol dehydrogenase assay kit and a pyruvate decarboxylase test kit (Nanjing Jiancheng Bioengineering Institute).

The field experiments were conducted at the Zhangbang Village, Dajin Town, Wuxue County, Hubei Province, China, in May 2016. In case of seed floating or stress from waterlogging conditions when heavy rains occurred soon after direct sowing, 20% (w/w) of CaO_2_ content at a pelleting time of three in formulae 1 and 2, which were consistent with that in Experiment 3. The treatments were randomly arranged using a randomized block design with four replicates. The plot area was 5 m^2^. The water layer in each plot was kept 2–3 cm until the seed germination was stable. The germination of seeds was recorded on daily basis according to AOSA (1990) until a constant count was achieved. Germination percentage was taken as the ratio of the number of seeds germinated to the total number of seeds sown and is expressed as a percentage. At 9 DAS, 10 seedlings were randomly sampled from each plot to record their shoot and root length. The seedlings from each plot were then dissected into roots and shoots, and their fresh weight was recorded immediately.

### Statistical Analysis

Data were analyzed to confirm variability following analysis of variance using Statistix 9.0. In the experiment 1 & field experiment, we used one-way ANOVA, pelleting treatments as grouping factors, germination, root length, shoot length, root fresh weight, shoot fresh weight as response variables. In the experiments 2 & 3, we used three-way ANOVAs, with formulae, CaO_2_ contents and pelleting times as grouping factors, germination, root length, shoot length, root fresh weight, shoot fresh weight as response variables. But the naked seeds were not involved in three-way ANOVAs. The differences between treatments were separated using a Least Significance Difference (LSD) test at a 0.05 probability level.
